# Primary tumor resection or systemic treatment as palliative treatment for patients with isolated synchronous colorectal cancer peritoneal metastases in a nationwide cohort study

**DOI:** 10.1007/s10585-023-10212-y

**Published:** 2023-05-20

**Authors:** Anouk Rijken, Vincent C. J. van de Vlasakker, Geert A. Simkens, Koen P. Rovers, Felice N. van Erning, Miriam Koopman, Cornelis Verhoef, Johannes H. W. de Wilt, Ignace H. J. T. de Hingh

**Affiliations:** 1 Department of Surgery, Catharina Hospital, Eindhoven, the Netherlands; 2 Department of Research and Development, Netherlands Comprehensive Cancer Organization, Utrecht, the Netherlands; 3 Department of Medical Oncology, University Medical Centre Utrecht, Utrecht University, Utrecht, the Netherlands; 4 Department of Surgery, Erasmus Medical Centre, Rotterdam, the Netherlands; 5 Department of Surgery, Radboud University Medical Centre, Nijmegen, the Netherlands; 6 GROW- School for Oncology and Development Biology, Maastricht University, Maastricht, the Netherlands

**Keywords:** Peritoneal metastases, Colorectal cancer, Palliative primary tumor resection, Palliative systemic treatment

## Abstract

Limited data are available to guide the decision-making process for clinicians and their patients regarding palliative treatment options for patients with isolated synchronous colorectal cancer peritoneal metastases (CRC-PM). Therefore, the aim of this study is to analyze the outcome of the different palliative treatments for these patients. All patients diagnosed with isolated synchronous CRC-PM between 2009 and 2020 (Netherlands Cancer Registry) who underwent palliative treatment were included. Patients who underwent emergency surgery or curative intent treatment were excluded. Patients were categorized into upfront palliative primary tumor resection (with or without additional systemic treatment) or palliative systemic treatment only. Overall survival (OS) was compared between both groups and multivariable cox regression analysis was performed. Of 1031 included patients, 364 (35%) patients underwent primary tumor resection and 667 (65%) patients received systemic treatment only. Sixty-day mortality was 9% in the primary tumor resection group and 5% in the systemic treatment group (*P* = 0.007). OS was 13.8 months in the primary tumor resection group and 10.3 months in the systemic treatment group (*P* < 0.001). Multivariable analysis showed that primary tumor resection was associated with improved OS (HR 0.68; 95%CI 0.57–0.81; *P* < 0.001). Palliative primary tumor resection appeared to be associated with improved survival compared to palliative systemic treatment alone in patients with isolated synchronous CRC-PM despite a higher 60-day mortality. This finding must be interpreted with care as residual bias probably played a significant role. Nevertheless, this option may be considered in the decision-making process by clinicians and their patients.

## Introduction

Colorectal cancer (CRC) is one of the most common cancers worldwide, with a yearly incidence of almost two million cases [1]. Frequently, CRC has already metastasized at the time of diagnosis, with the peritoneum as the second most affected organ being present in approximately 23% of patients with metastatic CRC [2]. In one third of these patients these metastases are confined to the peritoneum [3, 4].

Treatment of CRC patients presenting with peritoneal metastases (PM) is challenging and depends on various factors including the condition of the patient, the presence of systemic metastases, symptoms of the primary tumor and extend of the peritoneal disease [5, 6]. A selected group of fit patients with limited peritoneal disease may undergo curative intent treatment such as cytoreductive surgery with or without hyperthermic intraperitoneal chemotherapy (CRS-HIPEC) [7]. Patients with a symptomatic primary tumor (e.g., obstruction or perforation) are usually treated with surgery in an emergency setting [8].

For fit patients that do not require emergency surgery and in whom curative intent treatment is not possible due to extensive disease, two palliative treatment options may be considered: resection of the primary tumor (with or without additional systemic treatment) or palliative systemic treatment only. Whether to resect an asymptomatic primary colorectal tumor in patients presenting with unresectable systemic metastases has been a highly debated issue for many years with various retrospective studies and recently published randomized trials reporting conflicting results [9–21].

However, it should be noted that both in these prospective trials and retrospective studies the vast majority of included patients suffered from liver metastases and/or lung metastases [9–21]. Patients with PM were either absent or represented only a very small proportion of the study population. Thus, these studies give no guidance regarding the treatment of patients with CRC and isolated PM. This is relevant as patients with colorectal cancer peritoneal metastases (CRC-PM) are known to have a different clinical outcome as compared to CRC patients with liver metastases or lung metastases with a markedly shorter survival [22]. This may be due to the observation that PM seem to respond less to systemic treatment as compared to other systemic metastases [23–25]. Therefore, the aim of the current study was to analyze the outcome of palliative primary tumor resection (with or without additional systemic treatment) and palliative systemic treatment only specifically in CRC patients with isolated synchronous PM who did not undergo emergency surgery or curative intent treatment.

## Materials and methods

### Data source

Data were extracted from the Netherlands Cancer Registry (NCR). The NCR registers all newly diagnosed malignancies in the Netherlands. Specially trained data managers of the NCR extract data on patient, tumor and treatment characteristics from the medical records. A yearly update of the vital status of patients is performed by linking the registry to the Dutch municipal administrative database, which contains information about all present, deceased and former inhabitants of the Netherlands. For the present study, the latest update was performed on January 31st, 2022. The International Classification of Diseases for Oncology (ICD-O) was used for the specification of the primary tumor location, location of synchronous metastases and for histological subtypes. The tumor node metastasis (TNM) classification was used for stage classification of the primary tumor, according to the edition valid at diagnosis. If pathological T or N stage was unknown, clinical T or N stage was used. The study is approved by the privacy review board of the NCR as well as the combined scientific committee of the NCR and Prospective Dutch ColoRectal Cancer Cohort (PLCRC) study of the Dutch Colorectal Cancer Group (DCCG).

### Study population

All CRC patients with synchronous metastases diagnosed between 2009 and 2020 were evaluated. In patients with multiple primary tumors, the tumor which was first diagnosed was included or, if simultaneously diagnosed, the tumor with the highest TNM stage was included. Patients with extraperitoneal metastases were excluded. Patients were also excluded if they had a primary tumor in the appendix or a neuroendocrine primary tumor. In addition, patients were excluded if they had undergone curative intent treatment such as CRS-HIPEC, debulking surgery or metastasectomy, if they had only received best supportive care, if the primary tumor was resected after initial systemic treatment or neo-adjuvant chemoradiotherapy or if the treatment was unknown. The NCR records whether the primary tumor resection was performed in an elective setting or in an emergency setting. Patients who underwent an emergency resection were excluded. If no data regarding the clinical indication for surgery was registered, patients who had undergone surgery within 5 days after their initial diagnosis were considered to be emergency resection rather than primary tumor resection and were excluded.

### Treatment allocation

For all analyses, treatment strategies were categorized as follows:

1. Upfront palliative primary tumor resection with or without additional systemic treatment, comprising different types of resections (i.e., hemicolectomy, ileocecal resection, transverse colon resection, sigmoid resection, (sub)total colectomy, low anterior resection and rectum amputation).
2. Palliative systemic treatment only.

### Primary outcome

The primary outcome was overall survival (OS), compared between patients in the palliative primary tumor resection group and patients in the palliative systemic treatment group. Median OS was defined as the interval between date of diagnosis of CRC until date of death or loss to follow-up. Patients were censored if they were alive on January 31st, 2022.

### Patient- and tumor characteristics

The location of the primary tumor was categorized according to the following sites: (1) right-sided colon (C18.0, C18.2-18.4: cecum, ascending colon, hepatic flexure, transverse colon); (2) left-sided colon (C18.5-18.7: splenic flexure, descending colon and sigmoid); and (3) rectum (C19.9-20.9: rectosigmoid and rectum). Primary tumor histology was categorized into the following subtypes: (1) adenocarcinoma (8000, 8010, 8020, 8140, 8144, 8210, 8211, 8220 8255, 8261, 8262, 8263, 8560); (2) mucinous adenocarcinoma (8480, 8481); and (3) signet ring cell carcinoma (8490). The following ICD-O codes were considered PM: C16.0-C16.9, C17.0-C17.9, C18.0-C18.9, C19.9, C20.9, C21.8, C23.9, C26.9, C48.0-C48.8, C49.4-C49.5, C52.9, C54.3-C54.9, C55.9, C56.9, C57.0-C57.8, C66.9, C67.0-C67.9, C76.2. Any other ICD-O code was considered to reflect extraperitoneal metastases. Patient- and tumor characteristics included in this study are sex, age, primary tumor location, tumor histology, differentiation of primary tumor, T stage, N stage and period of diagnosis.

### Statistical analysis

Baseline characteristics of patients in the primary tumor resection group were compared to patients in the palliative systemic treatment group. Categorical variables were compared using χ^2^-test and presented as a No. (%), and continuous variables were compared with the unpaired *t*-tests and presented as mean (standard deviation [SD]). Missing data were excluded from comparative analyses. Sixty-day mortality was compared between patients in the palliative primary tumor resection group and the palliative systemic treatment group by using the χ^2^-test. Median OS of patients in the palliative primary tumor resection group and patients in the palliative systemic treatment group was estimated with the Kaplan Meier method and compared with the log-rank test.

Univariable cox regression analyses were performed to assess the association between palliative primary tumor resection and OS and to identify whether the following factors were associated with OS: sex, age, primary tumor location, tumor histology, tumor differentiation, T stage, N stage, period of diagnosis and the presence of a stoma. Subsequently, variables with a p-value lower than *0.10* in the univariable analyses, were combined in a multivariable cox regression model. To prevent overfitting, a minimum of 10 events per degree of freedom was used as limit for the number of variables of the multivariable model.

Finally, a subgroup analysis was performed in patients who underwent primary tumor resection. This subgroup analysis included uni- and multivariable cox regression analyses to identify factors associated with OS within this subgroup.

All tests were two-sided and a p-value lower than *0.05* was considered statistically significant. All analyses were performed with SAS statistical software (SAS system 9.4, SAS Institute, Cary, NC, United States).

## Results

### Study population

**Fig. 1:**
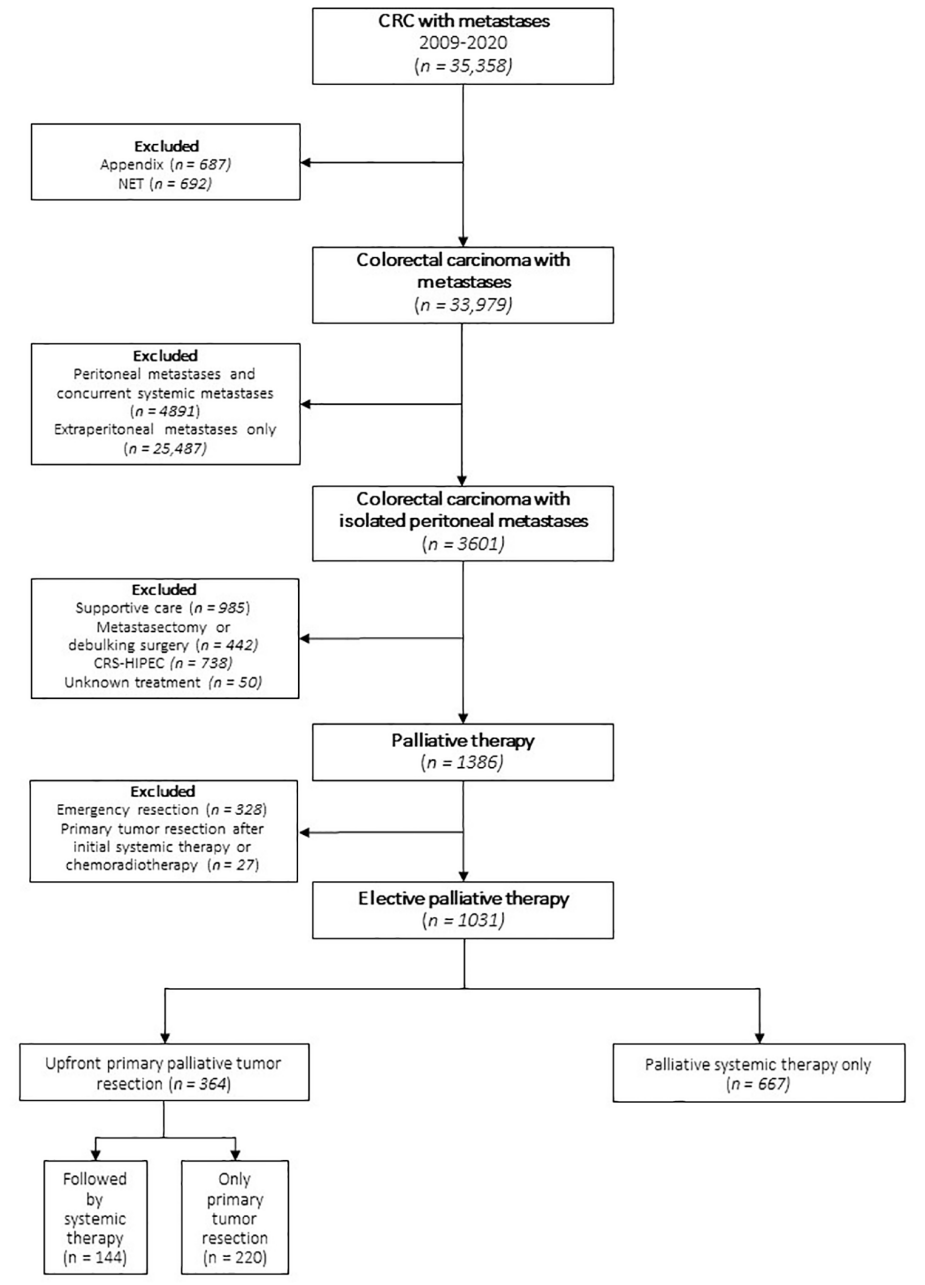
Patient selection and treatment allocation *CRC* indicates colorectal cancer; *NET* indicates neuroendocrine tumor; *CRS-HIPEC* indicates cytoreductive surgery and hyperthermic intraperitoneal chemotherapy

In total, 33.979 patients were diagnosed with metastasized CRC between 2009 and 2020. Of these patients, 8492 (25%) had synchronous PM of whom 3601 (11%) without concurrent extraperitoneal metastases. Of this latter group, 2215 (62%) patients were excluded because they had undergone curative treatment, best supportive care or an unknown treatment modality. An additional 328 patients undergoing primary tumor resection within five days of diagnosis of their primary CRC were excluded and 27 patients were excluded because their primary tumor was resected after initial systemic treatment or neo-adjuvant chemoradiotherapy. The remaining 1031 patients were included in this study, of whom 364 (35%) underwent primary tumor resection. In the palliative systemic treatment group (n = 667/1031, 65%), patients were exclusively treated with palliative systemic treatment. The primary tumor resection group (n = 364) comprised of 220 (60%) patients who underwent primary tumor resection only and 144 (40%) patients who underwent primary tumor resection followed by additional systemic treatment (Fig. 1).

In patients who underwent a primary tumor resection followed by additional systemic treatment (n = 144), 126 patients (88%) received chemotherapy only and 18 patients (12%) received both chemotherapy and targeted therapy. Details regarding the prescribed regimens were registered in 46 patients (32%). In these patients, capecitabine with oxaliplatin (CAPOX) (n = 20) and capecitabine monotherapy (n = 17) were the most used chemotherapeutic regimens.

In the palliative systemic treatment group (n = 667), 549 patients (82%) received chemotherapy only, 5 patients (1%) received targeted therapy only and 113 patients (17%) received both chemotherapy and targeted therapy. Details regarding the prescribed regimens were registered in 345 patients (52%), capecitabine with oxaliplatin (CAPOX) (n = 169), capecitabine monotherapy (n = 89) and 5-fluorouracil/leucovorin with oxaliplatin (FOLFOX) (n = 58) being the most used chemotherapeutic regimens. Panitumumab (n = 19) was the most used targeted therapy in these patients.

Older age, a right-sided tumor, a T4 tumor stage, positive lymph nodes and primary tumor diagnosis between 2009 and 2012 were more frequently present in patients who underwent palliative primary tumor resection than in those who received palliative systemic treatment (Table 1).

**Table 1:** Comparison of baseline characteristics between treatment groups

	Palliative primary tumor resection (n = 364)	Palliative systemic therapy (n = 667)	*P value* ^*a*^
**Sex, No. (%)**			
Male	186 (51)	384 (58)	*0.05*
Female	178 (49)	283 (42)	
**Age at diagnosis, mean (SD)**	72 (11)	65 (12)	*< 0.001*
**Primary tumor location, No. (%)**			
Right colon	234 (60)	325 (49)	
Left colon	114 (31)	257 (39)	*< 0.001*
Rectum	16 (4)	85 (13)	
**Tumor histology, No. (%)**			
Adenocarcinoma	230 (63)	389 (58)	
Mucinous adenocarcinoma	94 (26)	181 (27)	*0.19*
Signet ring cell carcinoma	40 (11)	97 (15)	
**Tumor differentiation, No. (%)**			
Well/moderately	165 (45)	119 (18)	
Poor/undifferentiated	140 (38)	128 (19)	*0.17*
Missing data	59 (16)	420 (63)	
**T stage, No. (%)**			
T1 – T3	138 (38)	173 (26)	
T4	224 (62)	192 (29)	*0.01*
Missing data	2 (1)	302 (45)	
**N stage, No. (%)**			
N0	51 (14)	214 (32)	
N1/N2	311 (85)	251 (38)	*< 0.001*
Missing data	2 (1)	202 (30)	
**Period of diagnosis, No. (%)**			
2009–2012	192 (53)	212 (32)	
2013–2016	109 (30)	246 (37)	*< 0.001*
2017–2020	63 (17)	209 (31)	
**Stoma, No. (%)**			
Yes	83 (23)	140 (21)	*0.50*
No	281 (77)	527 (79)	

^a^Missing data were not included in the comparative analyses; Percentages might not add up to 100% due to rounding; *SD* indicates standard deviation

### Survival

**Fig. 2:**
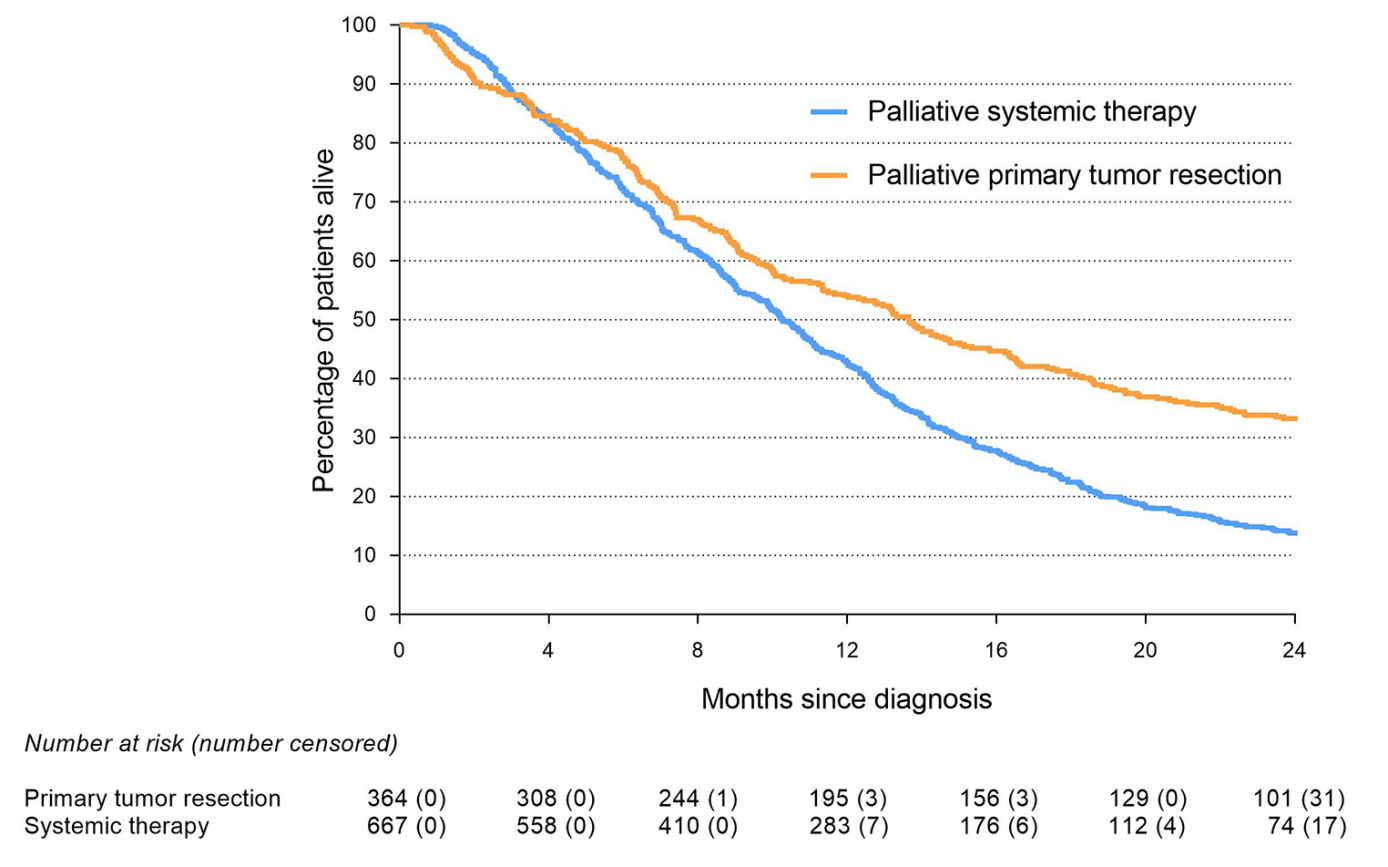
Overall survival of palliative primary tumor resection group and palliative systemic therapy group (Log-rank: <0.001) *PTR* indicates primary tumor resection; *ST* indicates systemic therapy

Sixty-day mortality was 9% in the primary tumor resection group and 5% in the palliative systemic treatment group (*P* = 0.007). Two-year survival was 32% in the primary tumor resection group and 14% in the palliative systemic treatment group (*P* < 0.001). The median OS was 13.7 (interquartile range [IQR] 6.4–29.4) months in the primary tumor resection group and 10.3 (IQR 5.5–17.0) months in the palliative systemic treatment group (*P* < 0.001) (Fig. 2). If a primary tumor resection was followed by systemic therapy, median OS was 18.0 months (IQR 8.9–33.4).

Univariable and multivariable analysis showed that primary tumor resection was significantly associated with improved OS (adjusted hazard ratio [aHR], 0.68; 95% CI, 0.57–0.81; *P* < 0.001). Factors that were associated with worse OS included a signet ring cell carcinoma histology (aHR, 1.38; 95% CI, 1.13–1.68; *P* = 0.001), a poor differentiated tumor (aHR, 1.49; 95% CI, 1.24–1.78; *P* < 0.001), a T4 tumor stage (aHR, 1.25; 95% CI, 1.07–1.46; *P* = 0.005) and nodal involvement (aHR, 1.28; 95% CI, 1.09–1.51; *P* < 0.001]) (Table 2).

**Table 2:** Uni- and multivariable cox regression analyses for overall survival of the entire study cohort

			Univariable analyses			Multivariable analysis		
	Total group (n = 1031)	Median OS (months)	HR	95% CI	*P value*	aHR	95% CI	*P value*
**Palliative therapy**					< 0.001			
Primary tumor resection	364	13.7	0.61	0.53–0.70		0.68	0.57–0.81	< 0.001
Systemic therapy	667	10.3	Ref	Ref		Ref	Ref	Ref
**Sex**					0.30			
Male	570	10.7	Ref	Ref		-	-	-
Female	461	11.3	0.94	0.82–1.06		-	-	-
**Age at diagnosis**	-	-	1.00	1.00-1.01	0.64	-	-	-
**Primary tumor location**					0.40			
Right colon	559	11.0	Ref	Ref		-	-	-
Left colon	371	11.5	0.99	0.86–1.13		-	-	-
Rectum	101	10.4	1.14	0.93–1.41		-	-	-
**Tumor histology**					< 0.001			
Adenocarcinoma	619	12.2	Ref	Ref		Ref	Ref	Ref
Mucinous adenocarcinoma	275	10.0	1.14	0.98–1.35		1.08	0.93–1.26	0.32
Signet ring cell carcinoma	137	9.1	1.53	1.28–1.82		1.38	1.13–1.68	0.001
**Tumor differentiation**					< 0.001			
Good/moderately	284	15.8	Ref	Ref		Ref	Ref	Ref
Poor/undifferentiated	268	8.1	1.64	1.36–1.99		1.49	1.24–1.78	< 0.001
Missing data	479	10.5	1.68	1.46–1.95		1.24	1.03–1.48	0.02
**T stage**					< 0.001			
T1 – T3	311	13.1	Ref	Ref		Ref	Ref	Ref
T4	416	11.0	1.24	1.06–1.45		1.25	1.07–1.46	0.005
Missing data	304	9.0	1.74	1.48–2.04		1.42	1.18–1.71	< 0.001
**N stage**					< 0.001			
N0	265	12.7	Ref	Ref		Ref	Ref	Ref
N1/N2	562	10.8	1.08	0.92–1.25		1.28	1.09–1.51	0.003
Missing data	204	8.9	1.55	1.29–1.85		1.32	1.09–1.60	0.005
**Period of diagnosis**					0.35			
2009–2012	404	10.7	Ref	Ref		-	-	-
2013–2016	355	10.7	1.07	0.93–1.24		-	-	-
2017–2020	272	11.3	0.95	0.81–1.12		-	-	-
**Stoma**					0.53			
Yes	223	10.3	Ref	Ref		-	-	-
No	808	11.2	0.95	0.82–1.11		-	-	-

*OS* indicates overall survival; *HR* indicates hazard ratio; *CI* indicates cumulative incidence; *aHR* indicates adjusted hazard ratio; *Ref* indicates reference. Variables with *P* > 0.10 were not used in the multivariable cox regression analyses.

### Subgroup analyses in primary tumor resection group

In subgroup analyses of patients who underwent primary tumor resection, multivariable analysis showed that older age (aHR, 1.02; 95% CI, 1.01–1.03; *P* = 0.002), a signet ring cell carcinoma histology (aHR, 1.58; 95% CI, 1.08–2.31; *P* = 0.02), a poor differentiated tumor (aHR, 1.68; 95% CI, 1.31–2.15; *P* < 0.001), a T4 tumor stage (aHR, 1.46; 95% CI, 1.15–1.85; *P* = 0.002), nodal involvement (aHR, 1.87, 95% CI, 1.33–2.63; *P* < 0.001 ) and having a stoma (aHR, 0.67, 95%CI, 0.51–0.85; *P = 0.002*) were associated with worse OS (Table 3).

**Table 3:** Uni- and multivariable cox regression analyses for overall survival of patients who have undergone a palliative primary tumor resection

			Univariable analyses			Multivariable analyses		
	Total group (n = 364)	Median OS (months)	HR	95% CI	*P value*	aHR	95% CI	*P value*
**Sex**					0.15			
Male	186	13.0	Ref	Ref		-	-	-
Female	178	14.6	0.85	0.69–1.06		-	-	-
**Age**	-	-	1.01	1.00-1.02	0.05	1.02	1.01–1.03	0.002
**Primary tumor location**					0.63			
Right colon	234	12.2	Ref	Ref		-	-	-
Left colon	114	16.6	0.90	0.72–1.12		-	-	-
Rectum	16	14.0	0.94	0.57–1.54		-	-	-
**Tumor histology**					0.003			
Adenocarcinoma	230	14.4	Ref	Ref		Ref	Ref	Ref
Mucinous adenocarcinoma	94	14.0	1.13	0.89–1.45		1.15	0.88–1.50	0.31
Signet ring cell carcinoma	40	8.6	1.80	1.28–2.52		1.58	1.08–2.31	0.02
**Tumor differentiation**					< 0.001			
Good/moderate	165	20.3	Ref	Ref		Ref	Ref	Ref
Poor/undifferentiated	140	8.5	1.71	1.32–2.21		1.68	1.31–2.15	< 0.001
Missing data	59	11.7	1.54	1.15–2.07		1.41	1.00-1.99	0.05
**T stage**					< 0.001			
T1-T3	138	16.5	Ref	Ref		Ref	Ref	Ref
T4	224	11.7	1.39	1.11–1.75		1.46	1.15–1.85	0.002
Missing data	2	3.7	8.47	3.40–21.10		7.18	0.44-118.06	0.17
**N stage**					< 0.001			
N0	51	25.8	Ref	Ref		Ref	Ref	Ref
N1/N2	311	13.2	1.72	1.26–2.36		1.87	1.33–2.63	< 0.001
Missing data	2	4.4	9.05	4.99–16.41		3.13	0.19–51.14	0.42
**Period of diagnosis**					0.36			
2009–2012	192	13.7	Ref	Ref		-	-	-
2013–2016	109	12.3	1.14	0.90–1.46		-	-	-
2017–2020	63	16.6	0.91	0.66–1.24		-	-	-
**Stoma**					0.05			
Yes	83	9.5	Ref	Ref		Ref	Ref	Ref
No	281	14.6	0.77	0.59-1.00		0.67	0.51–0.85	0.002

*OS* indicates overall survival; *HR* indicates hazard ratio; *CI* indicates cumulative incidence; *aHR* indicates adjusted hazard ratio; *Ref* indicates reference. Variables with *P* > 0.10 were not included in the multivariable cox regression analyses.

## Discussion

In this nationwide observational cohort study of patients with isolated synchronous CRC-PM, primary tumor resection was associated with an improved OS when compared to palliative systemic treatment only (median 13.7 months vs. 10.3 months). However, primary tumor resection was associated with an increase in sixty-day mortality. Patients undergoing treatment with curative intent, patients undergoing best supportive care only and patients requiring emergency surgery were excluded in this study and therefore, the results from the current study apply to those in whom the choice whether to perform a palliative resection of the primary tumor could be considered in a non-emergency setting.

The role of primary tumor resection in the treatment of patients with unresectable synchronous metastatic CRC with an asymptomatic primary tumor has been a highly debated issue for many years [21, 26–28]. Various retrospective studies seem to suggest a survival benefit after primary tumor resection [9, 10, 12–15]. However, selection bias may be an important explanation for this finding with younger and fitter patients usually tending to undergo surgery instead of systemic treatment. To address this issue in a prospective manner, several randomized trials have been conducted over the recent past. The recently published randomized controlled iPACs trial (JCOG1007) showed that the OS of systemic metastatic CRC patients who underwent primary tumor resection followed by systemic treatment was comparable to that of patients treated with systemic treatment only (26.4 months versus 25.9 months, respectively), which was in line with recently presented results from the SYNCHRONOUS trial [19, 29, 30]. Also, the CAIRO4 trial (NCT01606098) recently published the short-term results and reported a significantly higher mortality after primary tumor resection as compared to systemic treatment only (11% vs. 3% respectively) in the first 60 days after randomization [18]. As such, both trials provide valid arguments for no longer removing the primary tumor in CRC patients with widespread systemic disease [27]. As a result, resection of an asymptomatic primary colorectal tumor in patients with systemic metastases is no longer advised in most clinical guidelines such as the National Comprehensive Cancer Network [31].

Up to 10% of patients with CRC will be diagnosed with PM during the course of their disease [3]. As such, the peritoneum is a very relevant metastatic site in CRC. In spite of this, patients with PM are usually underrepresented in clinical trials as PM are often not visible on radiological imaging that is required for response evaluation to treatment [32, 33]. Also, in both previously mentioned retrospective and prospective trials investigating the effect of primary tumor resection, patients with PM were virtually absent [9–15, 18–21].

As recent data suggests that PM almost exclusively derive from a specific molecular subtype of CRC, it is probably not appropriate to translate knowledge that has been obtained in trials, performed in patients with liver metastases and lung metastases, to clinical scenarios in which PM are involved [34]. One reason may be that the subtype that causes PM is known to be less sensitive to systemic treatment [23, 25]. Together with the typical clinical presentation of PM with frequent bowel obstructions resulting in malnourishment, this probably explains that the prognosis of patients with PM is markedly worse as compared to other metastatic sites. Therefore, it can be argued that surgical treatment may indeed be more effective in alleviating clinical symptoms in this specific patient category than treating chemo-resistant metastases with systemic treatment.

The present study reported a higher sixty-day mortality for patients in the primary tumor resection group (9%) than for patients in the palliative systemic treatment group (5%). This finding is in line with a recently published randomized controlled trial on this topic for patients with CRC and systemic metastases [18]. This increase in early mortality confirms that primary tumor resection in patients with systemic disease does not come without substantial risk in the early postoperative period. Regarding OS, older age, a signet ring cell carcinoma histology, a poor tumor differentiation, a T4 tumor stage, nodal involvement and having a stoma were associated with a worse survival within the primary tumor resection group. Early postoperative mortality and risk factors for decreased OS after surgical treatment should be taken into account when discussing treatment options in these patients.

Construction of a stoma was necessary in 21% of patients treated with systemic treatment. No significant difference in the number of stomas was observed as compared to the primary tumor resection group. This is important in the decision-making process as fear for a stoma may deter patients from undergoing primary tumor resection.

In this study, the proportion of patients who received chemotherapy alone or in combination with targeted therapy was comparable between the systemic treatment group and the primary tumor resection group. In both groups, CAPOX was the most frequently used chemotherapy regimen. Therefore, treatment with systemic chemotherapy is not expected to result in a significant difference in survival.

Patients who underwent primary tumor resection in the present study were significantly older, more frequently had a right-sided tumor and nodal involvement as compared to patients that received systemic treatment only. Although multivariable cox regression analyses aimed to correct for these confounders after which primary tumor resection remained associated with an improved OS, residual selection bias probably still plays an important role. Relevant in this respect is the fact that the extent of peritoneal disease was not known. It may be that patients with less extensive peritoneal disease were more prone to undergo a primary tumor resection, which may explain the more favorable outcome in these patients.

To our knowledge, this is the first nationwide population-based study to investigate the role of primary tumor resection in patients with isolated synchronous CRC-PM. The NCR provides highly accurate data on tumor and patients characteristics, strengthening the generalizability of the results [35]. However, the retrospective design is clearly a drawback of the current study as no data on extent of peritoneal disease, tumor biology (e.g., CMS subtype), mutational status, performance status, postoperative complications or toxicity of systemic therapy and clinical symptoms were available. The addition of these factors would have increased the accurateness of the multivariable model.

It is not likely that a randomized controlled trial will address the issue of primary tumor resection in CRC patients with PM in the near future. Therefore, in spite of its retrospective nature, we believe that the current study provides valuable information to guide decision making in current day clinical practice in this distinct and relevant category of metastatic patients.

## Conclusions

In this retrospective nationwide cohort of patients with isolated synchronous CRC-PM, primary tumor resection appeared to be associated with an improved OS in comparison to those who received only systemic treatment, despite an increased sixty-day mortality rate after surgery. These findings must be interpreted with care as residual bias is likely to have played a significant role. Nevertheless, this finding may be considered in the decision-making process by clinicians and their patients regarding the different palliative treatment options in this specific patient category.
